# Electrochemical and Surface Characterization of Nickel-Containing Orthodontic Archwires Under In Vitro and In Vivo Conditions

**DOI:** 10.3390/dj14090567

**Published:** 2026-09-04

**Authors:** Angelina Stoyanova-Ivanova, Velizar Georgiev, Petar Lilov, Todor Vlakhov, Laura Andreeva, Valeri Petrov, Mirela Georgieva, Jorge N. R. Martins

**Affiliations:** 1 G. Nadjakov Institute of Solid-State Physics, Bulgarian Academy of Sciences, 72 Tzarigradsko Chaussee, 1784 Sofia, Bulgaria; angelina@issp.bas.bg (A.S.-I.); peter.lilov@issp.bas.bg (P.L.); todor_vlakhov@issp.bas.bg (T.V.); 2 Department of Orthodontics, Faculty of Dental Medicine, Medical University of Sofia, St. G. Sofiiski Blvd., 1431 Sofia, Bulgaria; laura_andreeva@fdm.mu-sofia.bg (L.A.); v.petrov@fdm.mu-sofia.bg (V.P.); mirela.georgieva@fdm.mu-sofia.bg (M.G.); 3 Faculdade de Medicina Dentária, Universidade de Lisboa, 1600-277 Lisboa, Portugal; jnrmartins@edu.ulisboa.pt; 4 Grupo de Investigação em Bioquimica e Biologia Oral, Unidade de Investigação em Ciências Orais e Biomédicas (UICOB), Faculdade de Medicina Dentária, Universidade de Lisboa, 1600-277 Lisboa, Portugal; 5 Centro de Estudo de Medicina Dentária Baseada na Evidência (CEMDBE)—Cochrane Portugal, Faculdade de Medicina Dentária, Universidade de Lisboa, 1600-277 Lisboa, Portugal; 6 LIBPhys-FCT UID/FIS/04559/2013, Faculdade de Medicina Dentária, Universidade de Lisboa, 1600-277 Lisboa, Portugal

**Keywords:** corrosion, cyclic voltammetry, electrochemical impedance spectroscopy, in-vitro, in-vivo, nickel-containing orthodontic archwires, open circuit voltammetry, scanning electron microscopy

## Abstract

**Objectives:** To evaluate the corrosion behavior and surface characteristics of four nickel-containing orthodontic archwires (stainless steel (SS), superelastic nickel–titanium (NiTi), copper–nickel–titanium (CuNiTi), and multiforce NiTi) under unused, in vitro artificial saliva-immersed, and clinically used (in vivo) conditions. **Methods:** Rectangular SS, NiTi, CuNiTi, and multiforce NiTi archwires were analyzed in the following three conditions: as received, after one week of immersion in artificial saliva (pH 6.4), and after clinical use for 6–8 weeks. Corrosion behavior was assessed using cyclic voltammetry (CV), open-circuit voltammetry (OCV), and electrochemical impedance spectroscopy (EIS). Surface morphology was examined by scanning electron microscopy (SEM). **Results:** Corrosion behavior was dependent on archwire type and exposure condition. SS archwires exhibited reduced impedance response after clinical use, indicating passive-film degradation. In the clinically used NiTi specimen, pronounced electrochemical instability was observed, characterized by a deep OCV transient, slow repassivation, and SEM evidence compatible with localized pitting corrosion. In the CuNiTi specimens, minimal differences were observed between the unused and clinically used conditions, which may be consistent with stable passive-film integrity in these specimens. In the multiforce specimen, clinical use was associated with a higher impedance response than the unused and saliva-immersed conditions of that same specimen. In the saliva-immersed specimens, possible passive-film formation and a higher impedance response were observed relative to the other conditions of the same specimens, but these did not reproduce the electrochemical and morphological changes observed after clinical use. **Conclusions:** In vitro artificial saliva immersion does not reliably replicate in vivo aging of nickel-containing orthodontic archwires. Corrosion behavior evolves during clinical service in an archwire-specific manner, with NiTi archwires showing susceptibility to clinically induced surface degradation.

## 1. Introduction

Metallic orthodontic appliances comprise a variety of components, including brackets, archwires, molar bands, ligature wires, and springs, which are routinely exposed to the oral environment throughout orthodontic treatment [1,2]. Some of the most commonly used orthodontic appliances are dental archwires made of nickel-containing alloys, which are used to treat conditions related to tooth misalignment. A treatment duration of approximately 6 weeks is a well-established clinical protocol [3,4,5], although treatment periods may extend to 10 weeks or longer [6,7]. The archwire types include austenitic stainless steel (SS), superelastic nickel–titanium (NiTi), thermoactivating copper–nickel–titanium (CuNiTi), and multiforce nickel–titanium archwires, all of which contain nickel in varying proportions. The clinical relevance of this lies in the fact that nickel is a metallic element frequently associated with hypersensitivity reactions during orthodontic treatment, and documented adverse responses include contact stomatitis, erythema multiforme, and gingival hypertrophy [8].

Archwires made from NiTi alloys usually contain 47–50 wt% nickel, with the remainder consisting of titanium, while SS alloys contain 8–25 wt% nickel and 17–25 wt% chromium, with the balance consisting of iron. CuNiTi archwires contain 5 wt% copper, 44 wt% nickel, and 51 wt% titanium, with traces of chromium (0.2–0.5 wt%) [1,9,10]. CuNiTi archwires are further differentiated into the following three variants based on their phase transformation (austenite-finish) temperature: 27 °C, 35 °C, and 40 °C, corresponding to superelastic, thermoelastic medium-force, and thermoelastic high-force delivery profiles, respectively [11,12]. The multiforce type of archwire is of particular clinical interest owing to its three distinct zones of progressive force release (frontal, premolar, and molar), with each zone delivering incrementally greater force, thereby enabling region-specific orthodontic loading [12,13].

Despite advances in orthodontic alloy manufacturing, which have led to the development of nickel-free alloys that retain or even improve the desired properties [14,15,16], corrosion remains a concern, as it results in the release of metal ions that may compromise both the physical performance of the appliance and the health of the surrounding tissues [17,18]. The oral environment presents a particularly challenging electrochemical milieu, as archwires are simultaneously exposed to fluctuating temperatures, variations in pH, enzymes, dental plaque, and ionic species, all of which modulate corrosion behavior [19]. Temperature effects are especially pertinent: intraoral temperatures may range from near 0 °C during cold food intake to 45 °C during the consumption of hot beverages, and it has been established that increasing temperature reduces the corrosion resistance of both SS and NiTi alloys, with NiTi demonstrating greater temperature sensitivity [20]. To simulate the oral environment while maintaining control over otherwise rapidly changing conditions, many studies utilize artificial saliva at different pH levels, in which the examined archwires are immersed for various periods of time [21,22].

While there are other methods available to evaluate and characterize the corrosion behavior of orthodontic alloys [23,24], electrochemical techniques have been used to demonstrate the superior corrosion resistance of NiTi compared with SS, attributable to the highly compact TiO_2_-rich passive layer formed on NiTi surfaces [25]. Such techniques include open-circuit voltammetry (OCV), cyclic voltammetry (CV), and electrochemical impedance spectroscopy (EIS), which are also widely applied because of their rapidity and high sensitivity. CV provides an indicator of corrosion tendency, OCV enables assessment of passive-film stability and susceptibility to pitting corrosion, and EIS yields mechanistic insight into passive-film properties, including polarization resistance and double-layer capacitance [17,20,26,27].

Comparatively little attention has been paid to the electrochemical examination of these types of archwires after use in an in vivo environment [10]. The present study employs OCV, CV, and EIS to examine the corrosion behavior of four nickel-containing archwire types (SS, superelastic NiTi, multiforce NiTi, and heat-activated CuNiTi archwires) in artificial saliva and after clinical use, with the aim of providing a qualitative electrochemical assessment of their corrosion behavior in both in vitro and in vivo environments.

## 2. Materials and Methods

### 2.1. Sample Selection and Preparation

The archwires studied in this work were all rectangular, with dimensions of 0.016 × 0.022 inches, and consisted of stainless steel (SS) archwires (Ortho-byte, Wilmington, DE, USA), superelastic nickel–titanium (NiTi) archwires (Ortho-byte, Wilmington, DE, USA), 35 °C copper–nickel–titanium (CuNiTi) thermoactivated archwires (Ormco, CA, USA), and multiforce NiTi archwires (GC Orthodontics Europe GmbH, Breckerfeld, Germany). All used archwires were clinically used in conjunction with MBT 0.22 metal brackets (American Orthodontics, Sheboygan, WI, USA). The archwires were separated into the following 3 groups: unused, clinically used (the periods of use are presented in Table 1), and those immersed in artificial saliva at pH 6.4.

The samples immersed in artificial saliva were cleaned using ethanol and distilled water after removal from the saliva. The clinically used samples were collected after the stage of treatment in which they were used was finished and cleaned according to the following protocol: (I) first, the examined archwires were wiped with gauze or cotton soaked in 3% hydrogen peroxide to remove food residues; (II) next, fresh gauze or cotton was soaked in 95% ethanol, and the archwires were cleaned repeatedly until all visible debris had been removed; and (III) finally, to ensure complete decontamination of the archwires and eliminate microbial contaminants, including bacteria and viruses, the used archwires were sprayed with IsoRapid spray (Oro Clean Chemie AG, Fehraltorf, Switzerland), left to stand for 1 min, and then rinsed under running water. This disinfection is mandatory for every used archwire in order to avoid infection. After disinfection, the archwires were segmented.

The used archwires were applied in the orthodontic treatment of 4 Bulgarian patients (one per type of wire). Treatment began in March 2026 and the wires were removed in May 2026, except for the multiforce wires, which were removed in April 2026. The selection of clinically used archwires included in the study was based on the following criteria: (a) patients with fully developed permanent dentition (i.e., all permanent teeth had erupted); (b) patients presented with good oral health, without caries or gingivitis, thereby minimizing the potential influence of inflammatory processes on oral pH; (c) patients were selected to be non-smokers and to refrain from consuming acidic or otherwise potentially erosive beverages, according to their self-reported data; and (d) all patients were systemically healthy and were given the same instructions by the treating orthodontist to follow the same low-fluoride and non-acidic dietary regimen for the duration of the treatment. All orthodontic patients provided written informed consent, which is mandatory in healthcare facilities in Bulgaria. The samples have been accounted in Table 2.

**Table 2:** Samples accounting for each archwire type.

Archwire Type	Patients	Wires	Tested Segments	Electro- Chemical Measurements	SEM Fields Photographed
Stainless steel (SS)	1	2	3	3	3
Superelastic nickel–titanium (NiTi)	1	2	3	3	3
Copper–nickel–titanium (CuNiTi)	1	2	3	3	3
Multiforce NiTi	1	2	3	3	3
Total	4	8	12	12	12

For each archwire type, one patient provided the clinically used specimen; the unused and saliva-immersed segments were cut from a separate unused source wire of the same type. Each specimen was assessed by a single electrochemical measurement per condition and a single representative SEM field per condition.

The artificial saliva (383.8 mg Na, 759.91 mg K, 61.26 mg Ca, 1171.26 mg Cl, 149.5 mg inorganic P, 200 g CH_4_N_2_O, 2 g C_6_H_8_O_6_, and 30 g C_6_H_12_O_6_ in 1000 mL of dH_2_O; pH 6.4) was prepared according to previously published protocols employing the same formulation [29,30,31]. Samples of the studied archwires were immersed in this solution at room temperature (25 °C) for a period of one week. The one week period is intended to represent a controlled, shorter-term model rather than a direct reproduction of clinical aging and to reduce the likelihood of bacterial biofilm development in the artificial saliva. This specific artificial saliva formulation was chosen because its pH is comparable to that of normal human saliva under physiological conditions.

For the electrochemical measurements, a 0.5 M NaCl working solution was prepared by mixing deionized water (dH_2_O) and ACS reagent-grade NaCl powder purchased from Sigma-Aldrich (Burlington, MA, USA). Each specimen was tested in 40 mL of 0.5 M NaCl solution at room temperature (25 °C). NaCl was used as a standardized chloride-rich electrolyte for electrochemical characterization rather than as a simulation of saliva or the oral environment; its near-neutral pH (7) was chosen to reduce the likelihood of the electrolyte itself additionally affecting the studied specimens.

### 2.2. Methodology

One 2 cm piece was cut from the posterior region of both the unused and clinically used archwires, with the exception of the multiforce archwire, which was cut in half, and one complete half was used due to its three distinct force-delivery zones. An additional 2 cm piece from the posterior region of each unused archwire was cut and immersed in artificial saliva (pH 6.4) for seven days.

Electrochemical testing was performed using a Bio-Logic SP-200 potentiostat (Bio-Logic Science Instruments, Seyssinet-Pariset, France) with a measurement accuracy of <±1 mV ± 0.03% of reading. Cyclic voltammetry (CV) was conducted at a scan rate of 50 mVs^−1^ over a potential window of 0.0–0.6 V. A potential window of 0.0 to +0.6 V was selected to evaluate the electrochemical behavior of the studied archwires in 0.5 M NaCl because it encompasses the passive region of nickel-containing biomedical alloys while avoiding excessive anodic polarization, extensive transpassive dissolution or severe oxygen evolution, which could irreversibly alter the alloy surface [32,33]. Open-circuit voltammetry (OCV) was performed for 900 s, and electrochemical impedance spectroscopy (EIS) was carried out with an amplitude of 100 mV and a frequency range of 10 kHz to 1 MHz, using a three-electrode electrochemical cell. In this configuration, the archwire piece served as the working electrode, while a polished platinum plate functioned as the counter electrode. A saturated calomel electrode (SCE) was used as the reference electrode. This configuration is widely employed in electrochemical studies because it enables precise control and accurate measurement of the working-electrode potential independently of the applied current. Quantitative EIS parameter extraction was outside the scope of this qualitative study and was not performed.

The morphology of the measured samples was investigated using a digitalized Philips 515 (Philips, Amsterdam, the Netherlands) scanning electron microscope (SEM). A total of 2 cm pieces of the orthodontic archwires were cut and mounted on SEM stubs using conductive adhesive tape. The displayed SEM fields in the “Results” section were selected after each segment was first visually examined and a representative section was selected to be photographed. The SEM analysis was performed at an accelerating voltage of 8 kV.

## 3. Results

### 3.1. Cyclic Voltammetry

Cyclic voltammetry (CV) measures the current response as the electrode potential is swept back and forth across a defined potential window. A larger loop area in the resulting voltammogram indicates higher electrochemical activity, reflecting greater capacitive current and/or Faradaic reactions, and is therefore associated with a more reactive surface which can mean a less protective passive film. CV was performed for each of the four archwire types under all three experimental conditions.

**Figure 1:**
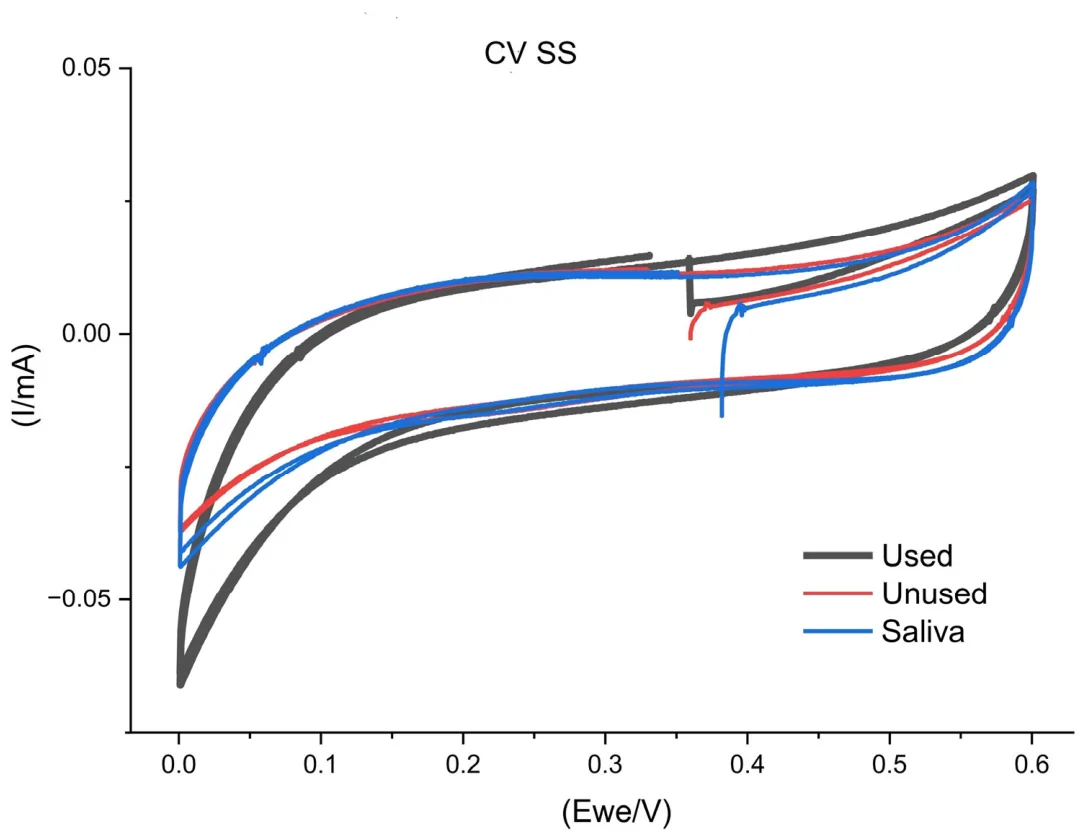
Cyclic voltammetry (CV) on stainless steel orthodontic archwire.

For the SS archwire (Figure 1), the used and unused SS specimens exhibited intermediate and broadly similar current responses, whereas the saliva-immersed specimen displayed a distinct electrochemical feature, a reduction-associated current inflection near 0.35 V, which can indicate partial passive-film dissolution or increased surface reactivity. The relatively larger loop area observed for the saliva-immersed specimen compared with the unused wire can indicate that exposure to artificial saliva promotes greater electrochemical activity and consequently reduced corrosion resistance in SS.

**Figure 2:**
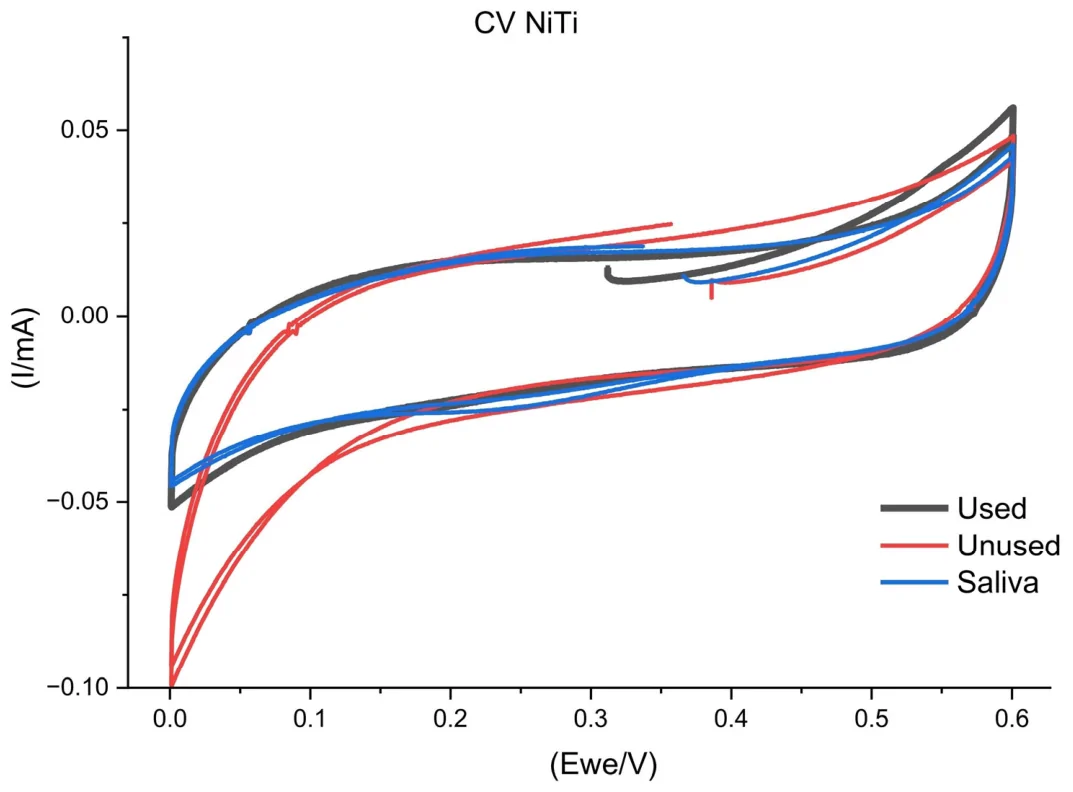
Cyclic voltammetry (CV) on NiTi orthodontic archwires.

For the NiTi archwire (Figure 2), the used and saliva-immersed specimens exhibited compact, closely overlapping loops with low capacitive currents, which can indicate stable passive-film behavior under these conditions. The unused specimens displayed an intermediate response.

**Figure 3:**
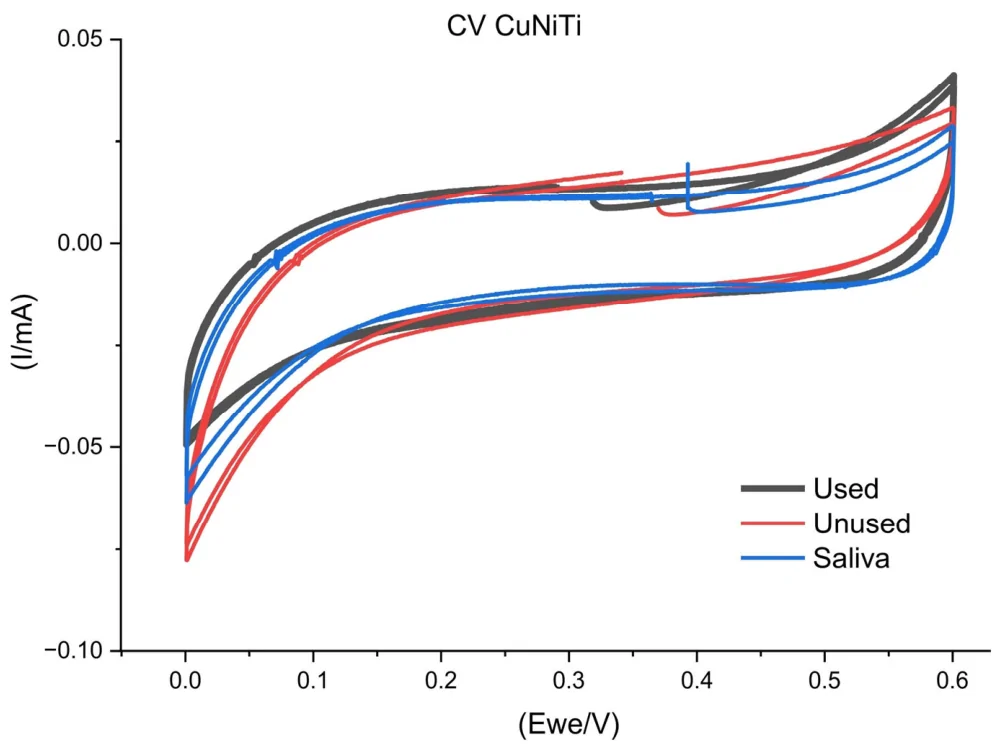
Cyclic voltammetry (CV) on CuNiTi orthodontic archwires.

When it comes to other conditions, while the unused wire remained within the same narrow range, the absence of significant inter-condition differences can suggest that the CuNiTi specimen (Figure 3) examined here maintained a stable and homogeneous surface across the exposure conditions tested.

**Figure 4:**
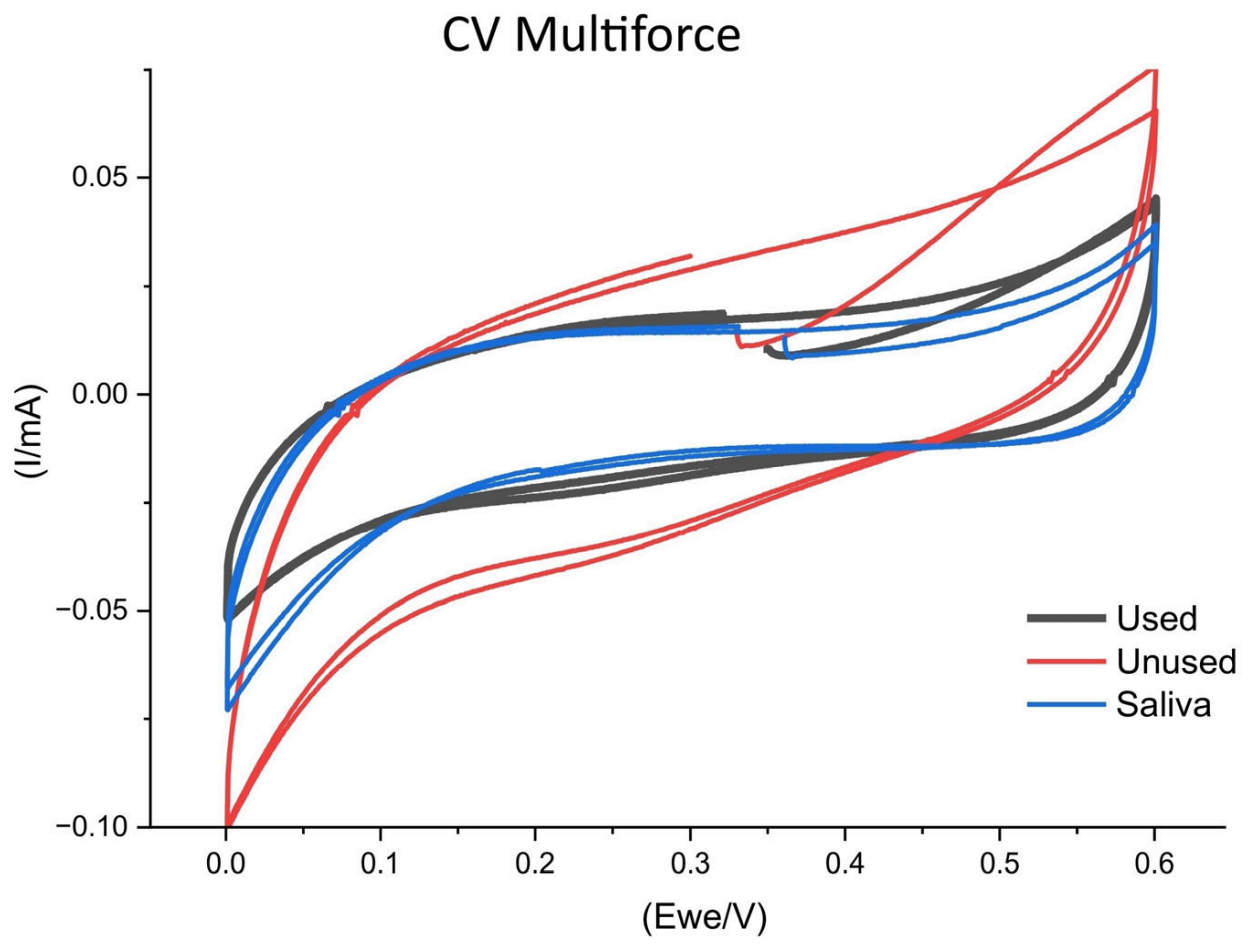
Cyclic voltammetry (CV) on multiforce orthodontic archwires.

For the multiforce archwire (Figure 4), the unused specimen produced a larger voltammetric loop than all other conditions, with anodic currents reaching approximately 0.075 mA and cathodic currents extending to approximately −0.10 mA. In contrast, the used and saliva-immersed specimens exhibited compact, closely overlapping loops, which can suggest that clinical use or immersion in artificial saliva promotes surface passivation and stabilization. This difference between the unused specimen and the exposed specimens suggests that the as-received multiforce archwire surface is initially more reactive, possibly due to the absence of a fully developed passive oxide layer, which forms upon exposure to oral or simulated oral environment.

Taken together, the CV results observed here indicate that the CuNiTi specimen showed closely similar electrochemical behavior across all three conditions tested, while the multiforce specimen showed a reduction in surface activity following exposure. The SS and NiTi specimens appeared to show more variation across the exposure conditions tested here, particularly immersion in artificial saliva.

### 3.2. Open-Circuit Voltammetry

Open-circuit voltage (OCV) measurements, which are indicative of surface passivation and corrosion resistance, were performed on all four archwire types under all experimental conditions. A more positive OCV that stabilizes rapidly can signify a more noble surface and can be interpreted as enhanced corrosion resistance.

**Figure 5:**
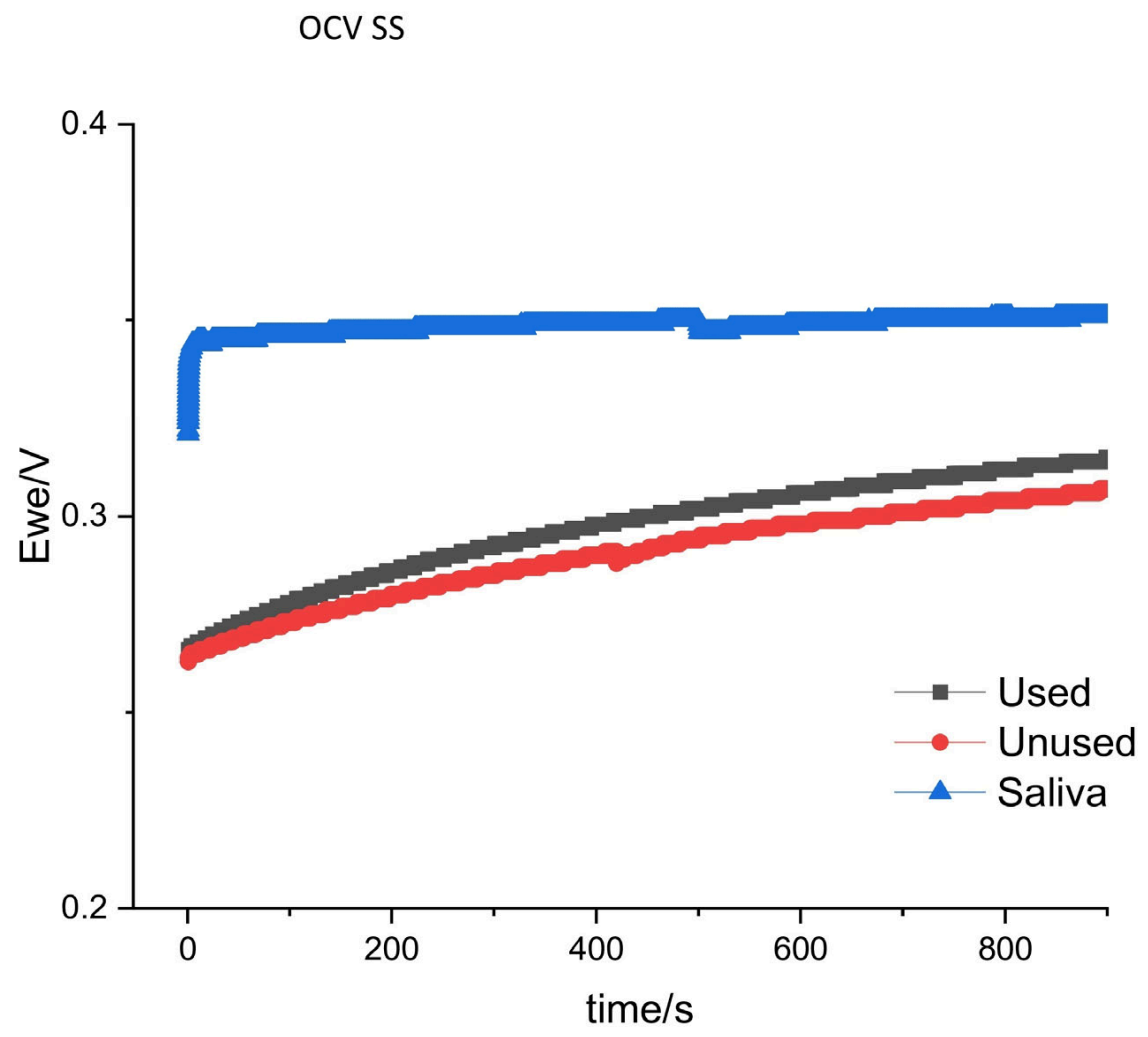
Open-circuit voltage (OCV) measurement on stainless steel orthodontic archwires.

SS archwires (Figure 5): The saliva-immersed SS specimen stabilized more rapidly and reached higher OCV values (~0.35 V) than the unused and clinically used SS specimens, which can be interpreted as passive-film formation in this specimen. The unused and clinically used specimens displayed similar, gradually increasing OCV values (~0.27–0.32 V), suggesting that clinical use did not significantly alter the initial electrochemical surface state.

**Figure 6:**
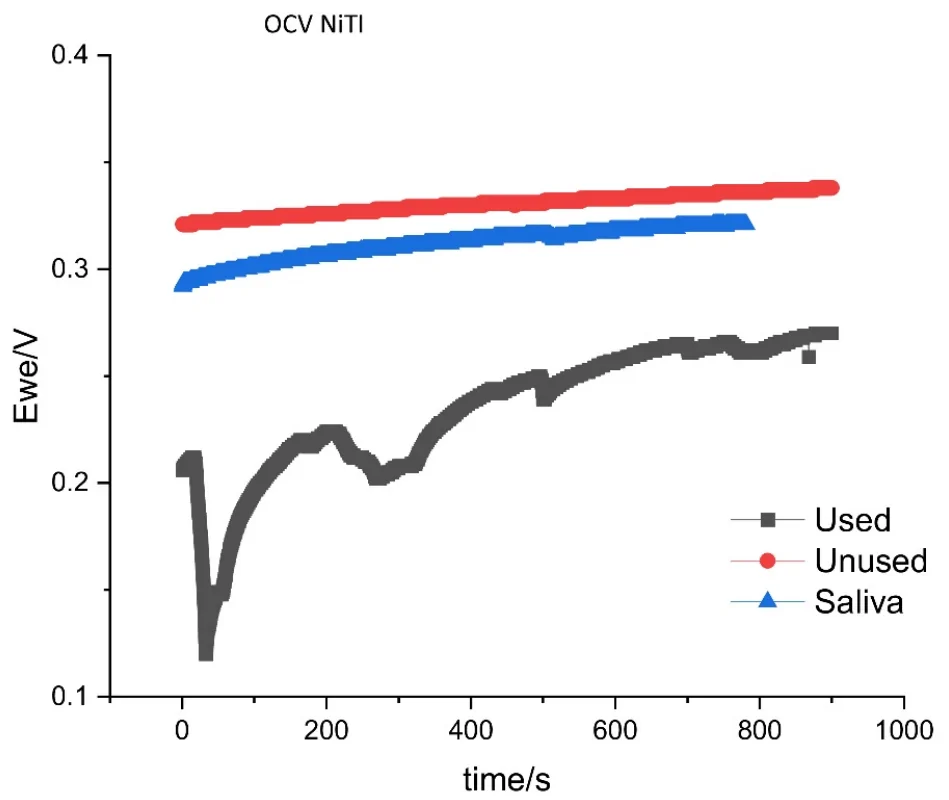
Open-circuit voltage (OCV) measurement on NiTi orthodontic archwires.

NiTi archwires (Figure 6): The unused NiTi specimen showed higher and more stable OCV values (~0.32–0.34 V) than the saliva-immersed and clinically used NiTi specimens. The saliva-immersed specimens closely followed this trend. In contrast, the clinically used NiTi specimens exhibited a possible pronounced cathodic transient, with the OCV decreasing to approximately 0.13 V before partially recovering to approximately 0.27 V. This behavior suggests significant surface disruption, which can mean reduced stability of the passive oxide layer in clinically aged NiTi archwire.

**Figure 7:**
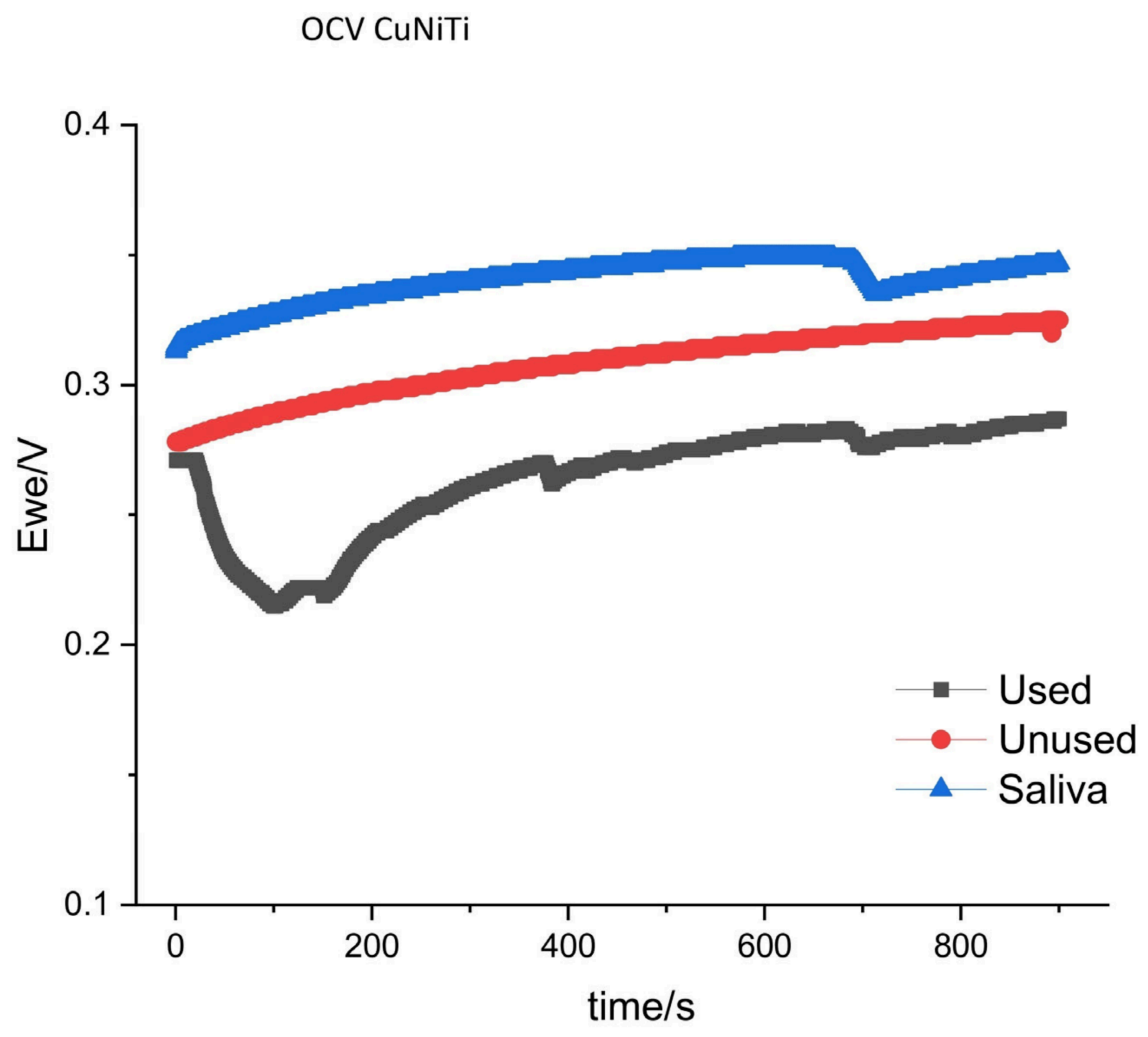
Open-circuit voltage (OCV) measurement on CuNiTi orthodontic archwires.

CuNiTi archwires (Figure 7): The saliva-immersed CuNiTi specimens attained high and stable OCV values (~0.31–0.35 V). The unused specimens exhibited a smooth increase in OCV over time. The clinically used CuNiTi specimens displayed an initial cathodic transient (from approximately 0.27 V to 0.22 V), followed by gradual recovery, indicating transient surface activation and possible partial breakdown of passive oxide film.

**Figure 8:**
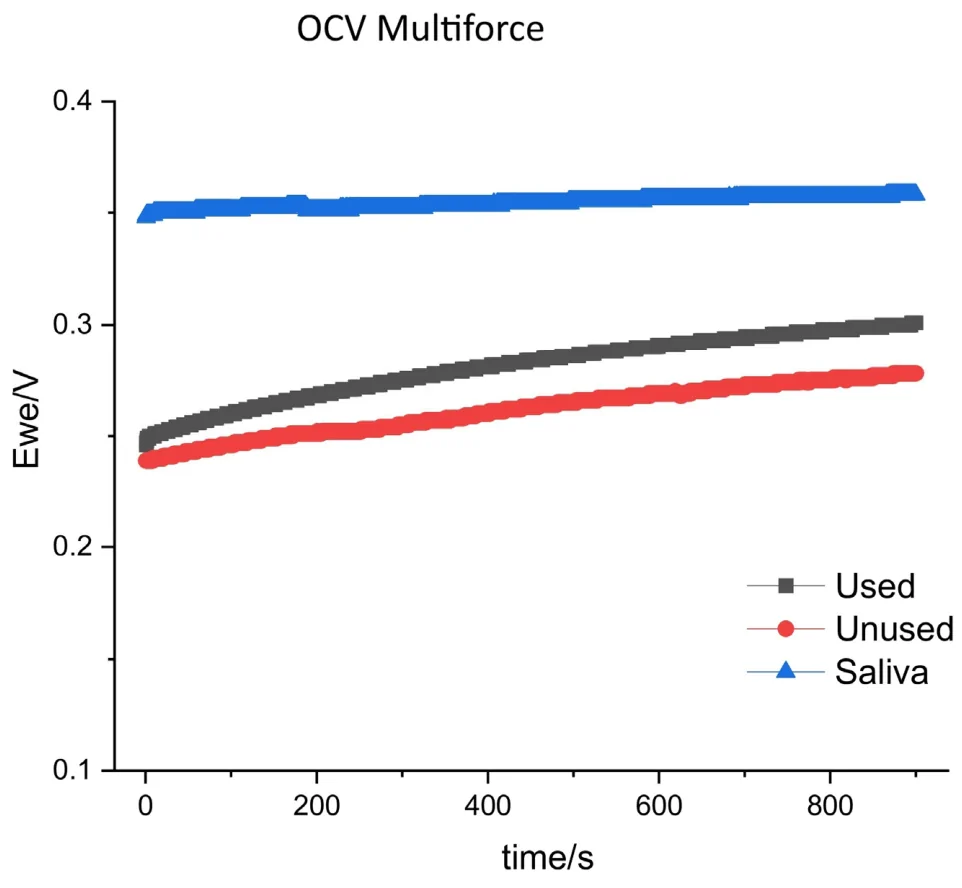
Open-circuit voltage (OCV) measurement on multiforce orthodontic archwires.

Multiforce archwires (Figure 8): The saliva-immersed multiforce specimen consistently showed higher and more stable OCV values (~0.35–0.36 V) than the unused and clinically used multiforce specimens. The unused and clinically used specimens displayed similar, gradually increasing OCV profiles (~0.24–0.30 V), suggesting that clinical use had minimal influence on their electrochemical surface state.

Overall, in the specimens examined in this work, saliva-immersed specimens consistently reached higher and more rapidly stabilized OCV values than the unused and clinically used specimens of the same archwire type, suggesting the development of passive surface films. The clinically used CuNiTi and NiTi specimens exhibited initial cathodic transients, with the NiTi specimen showing a more pronounced decrease and slower recovery than the CuNiTi specimen, indicating a possibly greater susceptibility of its clinically aged oxide layer to surface activation. In contrast, the clinically used SS and multiforce archwires exhibited OCV behavior closely resembling that of their unused counterparts.

### 3.3. Electrochemical Impedance Spectroscopy

Electrochemical impedance spectroscopy (EIS) measures the opposition to the current flow over a range of frequencies, providing information about the passive film formed on the archwire surface. In a Nyquist plot, the real component of the impedance, Re(Z), is plotted on the x-axis, while the imaginary component, −Im(Z), is plotted on the y-axis. A larger and more extended arc corresponds to a more protective surface film and a greater impedance response in the low-frequency region, which is consistent with a higher impedance response under the present test conditions. Notably, none of the archwire types produced a complete semicircle within the measured frequency range, indicating that the characteristic relaxation frequencies lie below the lower frequency limit of the measurement. This behavior is consistent with the presence of resistive passive oxide layers.

**Figure 9:**
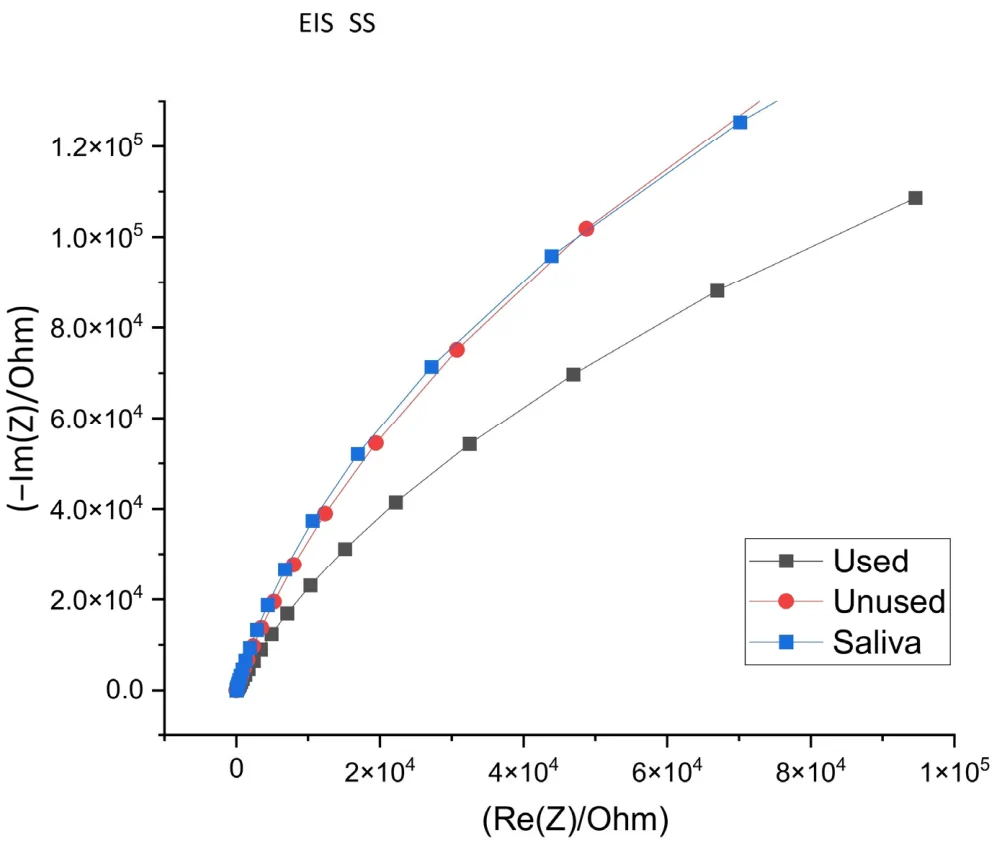
Electrochemical impedance spectroscopy (EIS) on stainless steel orthodontic archwires.

For the SS archwire (Figure 9), the unused and saliva-immersed specimens produced similar trajectories throughout the measured frequency range, both following steep, closely overlapping arcs that reached high Re(Z) and −Im(Z) values. In contrast, the clinically used specimen followed a lower trajectory. At equivalent Re(Z) values, its −Im(Z) values were consistently lower than those of the unused and saliva-immersed specimens. This divergence can indicate that clinical use reduced the overall impedance magnitude of the SS archwire relative to both the as-received and saliva-immersed states, suggesting that the passive surface oxide layer was partially compromised during the 8-week intraoral exposure period. The overlap of the unused and saliva-immersed curves further suggests that one week of immersion in artificial saliva at pH 6.4 did not significantly alter the passive-film characteristics of the SS archwire relative to its as-received condition.

**Figure 10:**
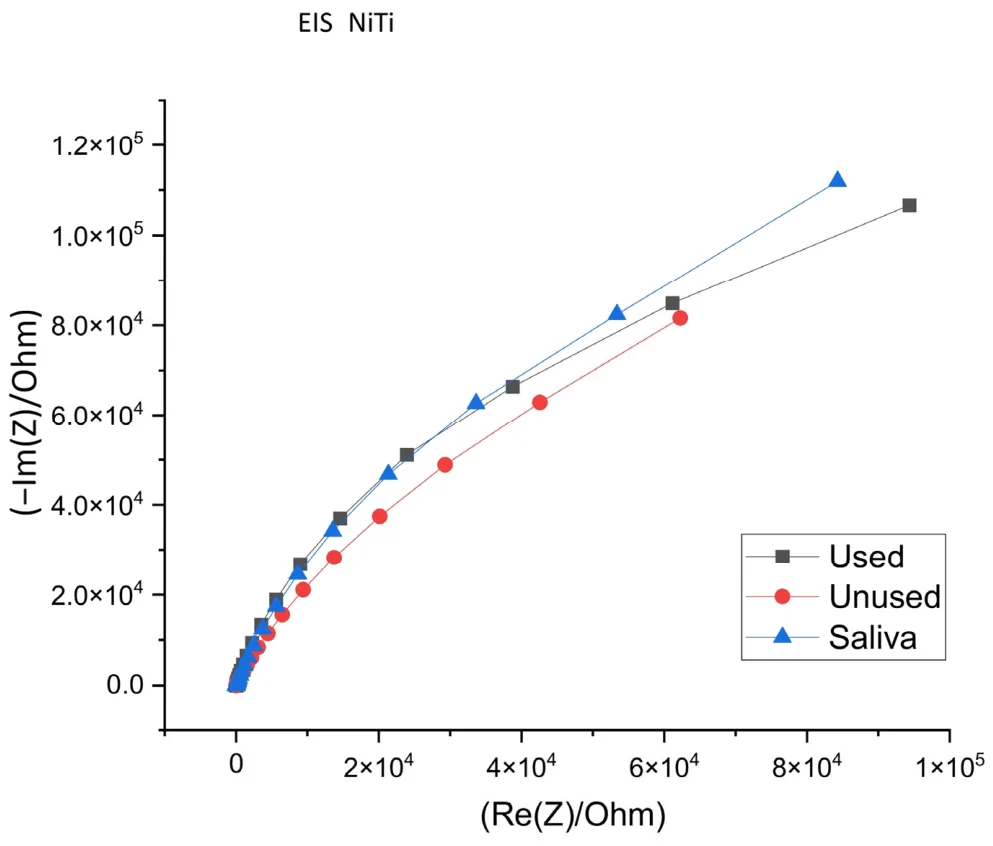
Electrochemical impedance spectroscopy (EIS) on NiTi orthodontic archwires.

For the NiTi archwire (Figure 10), the three conditions exhibited a more gradual separation of their impedance trajectories. The saliva-immersed specimen displayed a more extended arc, reaching higher −Im(Z) values at a given Re(Z) than the other two NiTi specimens throughout the plot, indicating a more protective passive film and higher impedance response in this specimen than in the unused and clinically used NiTi specimens. The clinically used specimen occupied an intermediate position, lying above the unused specimen at higher impedance values while remaining clearly below the saliva-immersed arc. The unused specimen exhibited the lowest impedance trajectory of the three. This ordering (saliva-immersed > clinically used > unused) differs from that observed for SS and suggests that, for NiTi, both clinical use and saliva immersion promote the development of a more protective passive film relative to the as-received archwire, with the artificial-saliva-immersed specimen showing a greater apparent enhancement in corrosion resistance than the other NiTi specimens studied here.

**Figure 11:**
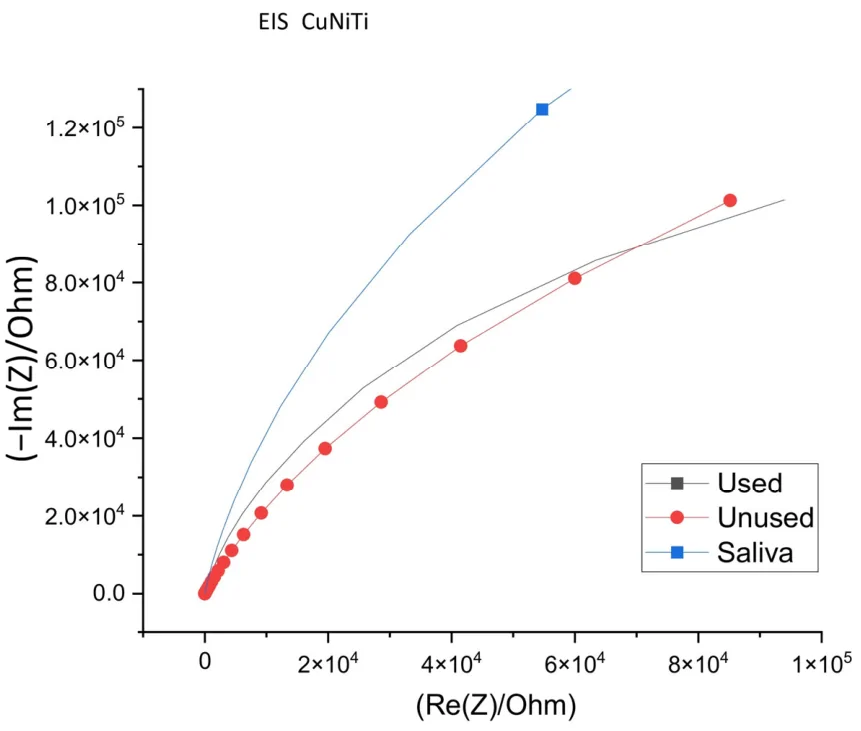
Electrochemical impedance spectroscopy (EIS) on CuNiTi orthodontic archwires.

For the CuNiTi archwire (Figure 11), pronounced divergence between conditions was observed in this specimen set. The saliva-immersed specimen exhibited a steep trajectory, reaching high −Im(Z) values at relatively low Re(Z) values and separating from the other two conditions from the beginning of the plot. In contrast, the clinically used and unused specimens followed closely grouped, more gradually increasing arcs that remained similar throughout the measured frequency range, with only a marginal offset between them. The marked elevation of the saliva-immersed arc relative to the clinically used and unused curves indicates that immersion in artificial saliva may have promoted the formation of a substantially more protective passive film on the CuNiTi surface, resulting in a substantially more extended impedance arc, indicative of a more protective surface state. The near-equivalence of the clinically used and unused trajectories suggests that eight weeks of clinical use had only a limited effect on the overall impedance characteristics of CuNiTi.

**Figure 12:**
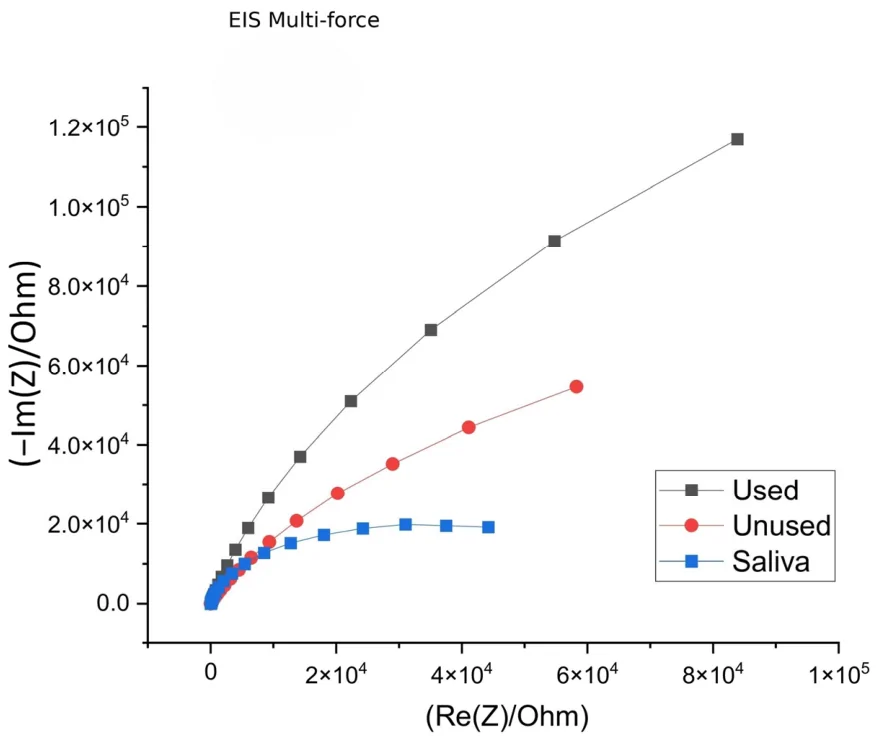
Electrochemical impedance spectroscopy (EIS) on multiforce orthodontic archwires.

For the multiforce archwire (Figure 12), the ordering of impedance responses was the inverse of that seen in CuNiTi. The clinically used specimen produced a more extended arc, reaching higher −Im(Z) values across the full Re(Z) range than the unused and saliva-immersed multiforce specimens, which is consistent with a higher impedance response in this specimen than in the other multiforce conditions tested under the present test conditions. The unused specimen occupied an intermediate position, with a moderately extended arc that fell clearly below the clinically used curve. The saliva-immersed specimen displayed the lowest impedance trajectory, with its arc flattening at comparatively low −Im(Z) values as Re(Z) increased, indicating that saliva immersion was associated with a reduction in the overall impedance magnitude of the multiforce archwire rather than an enhancement. This reversal (where the clinically used specimen showed higher impedance than the saliva-immersed specimen) was not observed for the other three archwire types studied and may reflect the multi-zone surface architecture of the multiforce archwire and its distinct response to the chemical environment of artificial saliva relative to the in vivo oral setting.

Taken together, the EIS results observed here reveal archwire-type-specific responses to the three exposure conditions in the specimens tested. The unused and saliva-immersed SS specimens were largely indistinguishable, while clinical use reduced the impedance of the SS specimen. The NiTi specimens showed a progressive increase in impedance from unused through clinically used to saliva-immersed conditions. The CuNiTi specimen was affected by saliva immersion, which produced a higher impedance response than the other CuNiTi conditions while leaving the clinically used and unused CuNiTi specimens comparable to each other. The multiforce specimen exhibited a pattern different from that of the other three archwire types tested, with the clinically used multiforce specimen showing higher impedance than the saliva-immersed multiforce specimen, underscoring the importance of in vivo exposure conditions in governing the corrosion behavior of this multiforce NiTi archwire.

### 3.4. Scanning Electron Microscopy

Scanning electron microscopy (SEM) was used to characterize the surface morphology of the four archwire types across the three experimental conditions.

**Figure 13:**
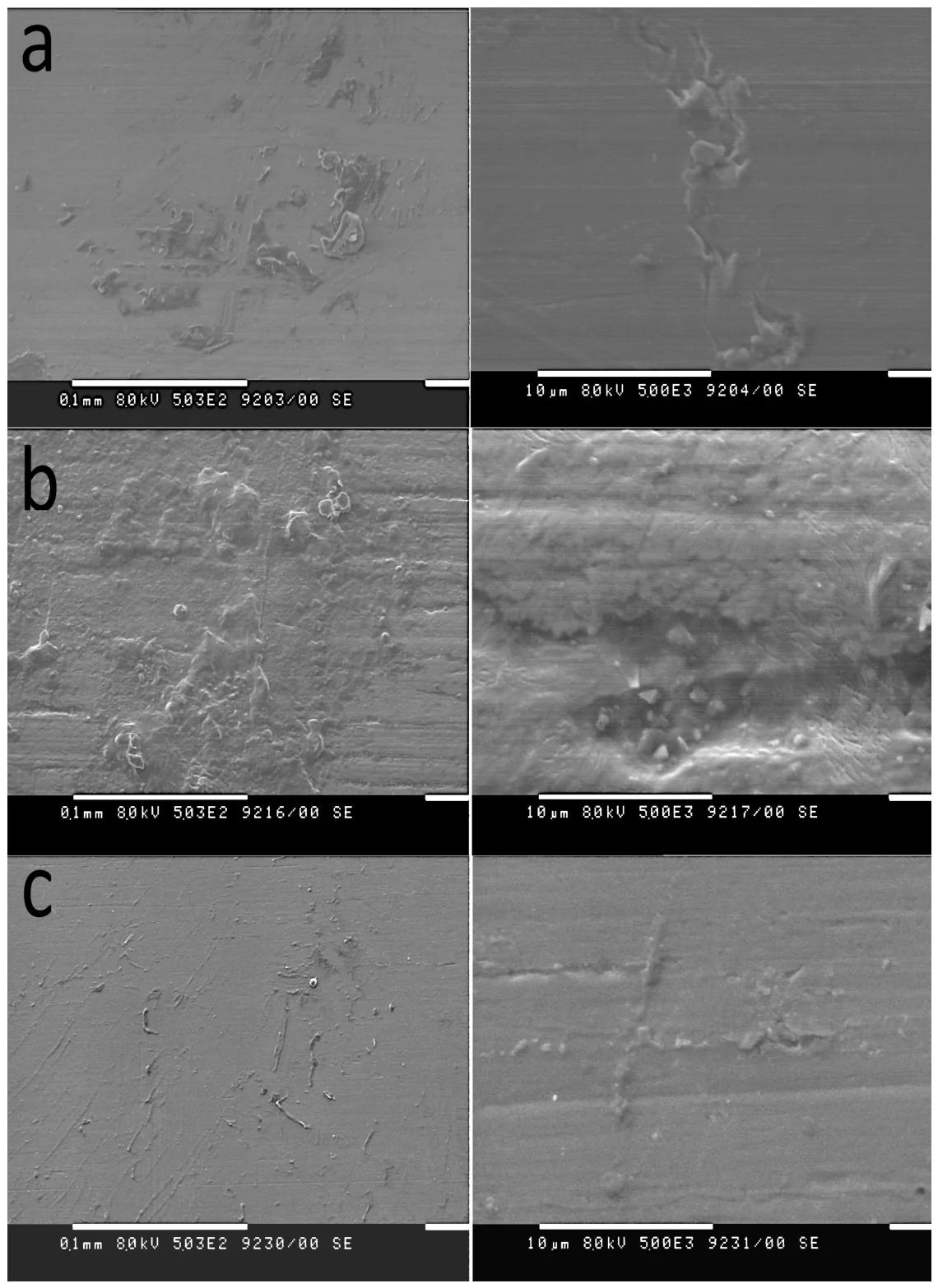
SEM micrographs of SS archwires—(**a**) unused, (**b**) used, (**c**) immersed in saliva (left image shows a ×503 magnification, right image shows ×5000 magnification).

Stainless steel archwires (Figure 13) exhibited manufacturing striations in the unused condition. Clinical use introduced granular deposits compatible with debris accumulation or corrosion products along pre-existing surface defects. Specimens immersed in artificial saliva exhibited a cleaner surface morphology than the unused and clinically used SS specimens, with this specimen devoid of significant particle accumulation. This observation is consistent with clinical exposure, rather than saliva immersion alone, being associated with localized surface contamination or corrosion-product deposition on this stainless steel specimen.

**Figure 14:**
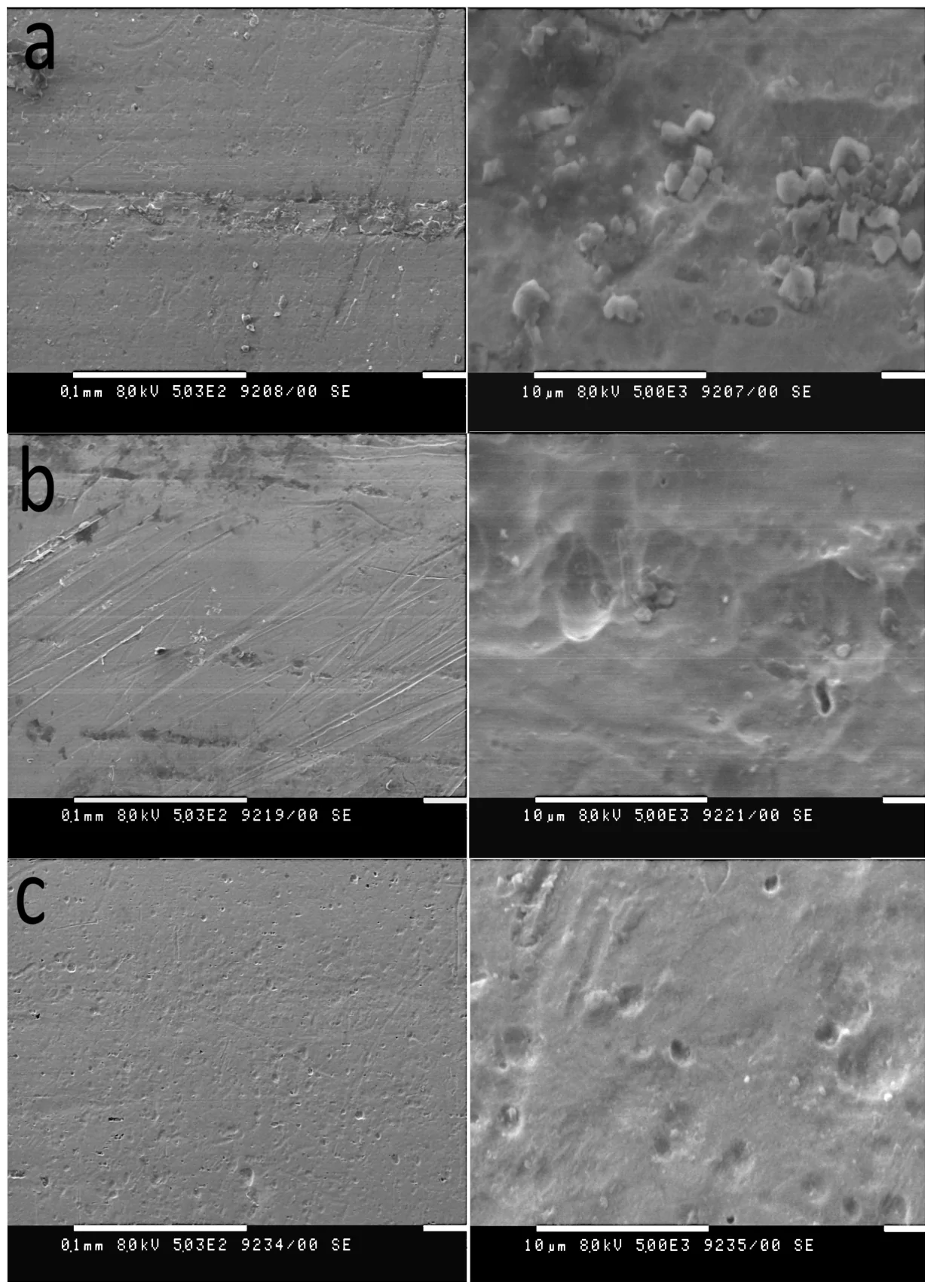
SEM micrographs of NiTi archwires—(**a**) unused, (**b**) used, (**c**) immersed in saliva (left image shows a ×503 magnification, right image shows ×5000 magnification).

Nickel–titanium archwires (Figure 14) in their unused state presented numerous large, irregularly shaped globular deposits and a rough, undulating surface. Clinically used specimens displayed distinct rounded pit-like depressions, which are compatible with localized pitting corrosion, along with pronounced diagonal striations indicative of mechanical abrasion. Specimens immersed in artificial saliva exhibited a comparatively smoother surface with scattered shallow depressions, lacking the pronounced deposits or pitting features observed in the other conditions. This observation possibly suggests a complex surface evolution for nickel–titanium archwires, with the as-received material exhibiting extensive deposits, clinical use inducing pitting corrosion and mechanical wear, and saliva immersion resulting in a more homogeneous surface morphology.

**Figure 15:**
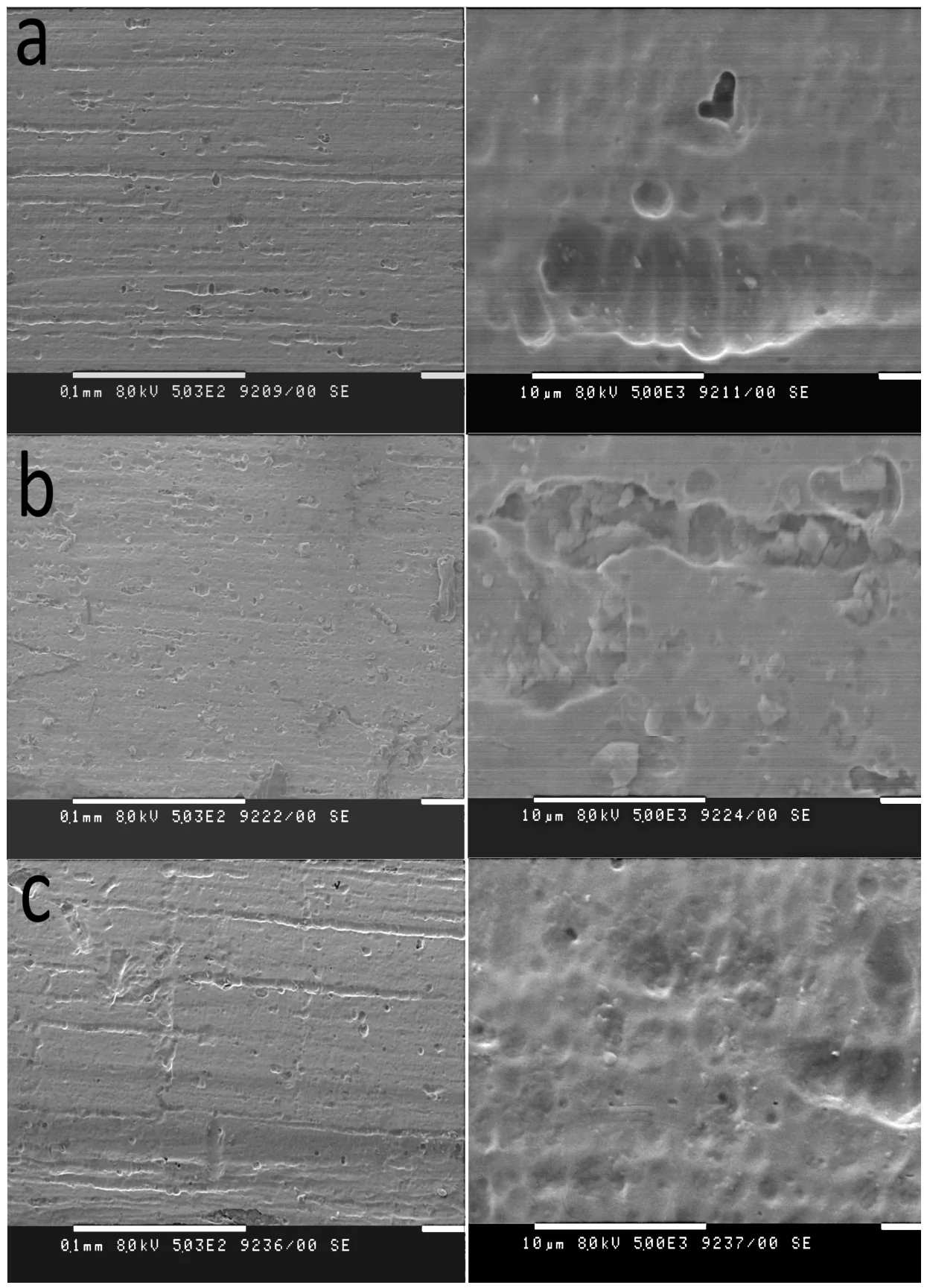
SEM micrographs of CuNiTi archwires—(**a**) unused, (**b**) used, (**c**) immersed in saliva (left image shows a ×503 magnification, right image shows ×5000 magnification).

Copper–nickel–titanium archwires (Figure 15), when unused, exhibited moderate surface complexity, including blister-like features and a deep pit, superimposed on longitudinal wire-drawing striations. Clinically used archwires demonstrated extensive, irregular material accumulation, compatible with the presence of substantial corrosion products or biological material. Specimens immersed in artificial saliva exhibited numerous rounded depressions and shallow pits but lacked the extensive deposits characteristic of clinical exposure. This suggests that clinical intraoral service induces significant material accumulation on CuNiTi archwires, a phenomenon that is not fully reproduced by artificial saliva immersion.

**Figure 16:**
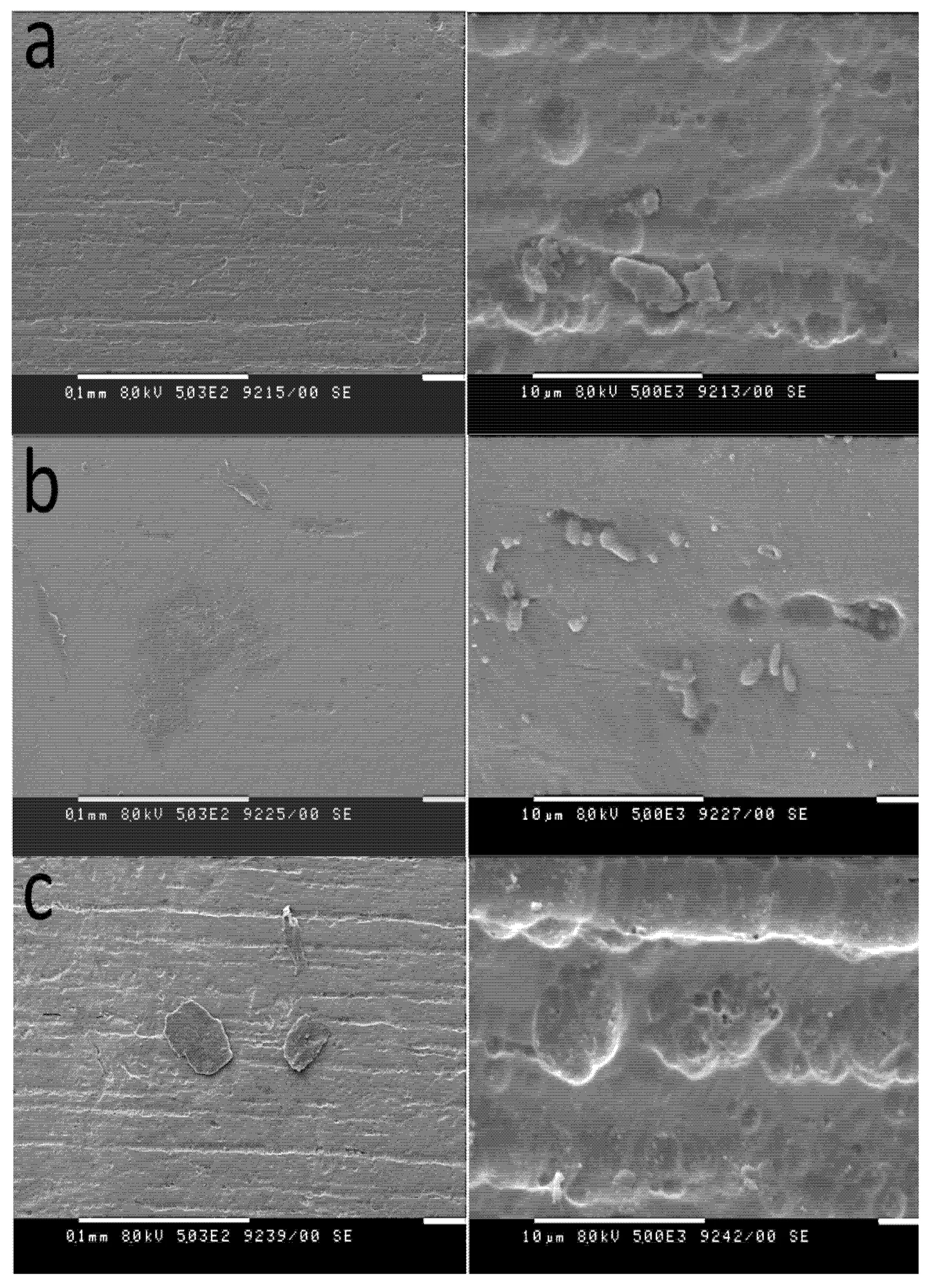
SEM micrographs of multiforce archwires—(**a**) unused, (**b**) used, (**c**) immersed in saliva (left image shows a ×503 magnification, right image shows ×5000 magnification).

For the multiforce archwire (Figure 16), the unused specimen exhibited a surface with scattered rounded pits or depressions of varying diameters, interspersed with larger, irregular, bright surface features in the lower field, against a broadly undulating background. The lower-magnification inset showed a relatively smooth surface with visible microstructural texture. The clinically used multiforce archwire (Figure 16b) displayed a partially modified surface at high magnification, with scattered globular and nodular deposits on an otherwise comparatively smooth surface, alongside visible superficial scratch marks.

Lower magnification similarly showed a predominantly smooth surface with isolated deposits. The saliva-immersed multiforce specimen (Figure 16c) exhibited a topographically complex appearance, with a prominent bright ridge feature extending along the upper portion of the high-magnification field, numerous crater-like depressions, and irregular surface relief. The lower-magnification inset revealed longitudinal grooves accompanied by scattered, flat, angular crystalline deposits, which is compatible with a surface reaction specific to this multiforce specimen’s composition and multi-zone architecture occurring during saliva immersion. Taken together, the multiforce SEM results reveal a pattern distinct from that observed for the other archwire types, wherein clinical use produces comparatively moderate surface alterations, whereas saliva immersion generates more pronounced topographical modification. This finding represents an inversion of the behavior observed for the other archwire types and is consistent with the distinct EIS response reported for the multiforce archwire.

## 4. Discussion

Taken together, the CV, OCV, and EIS results appear to indicate that the surface state of the archwires studied changes with both clinical use and immersion in artificial saliva and that the pattern of change differs by archwire type. Because the present data are comparative and qualitative in nature, these observations are offered as descriptive interpretations rather than firm conclusions.

Across the archwire types examined here, saliva-immersed specimens tended to show more positive OCV values and, in most cases, higher EIS impedance than the unused or clinically used specimens, which may point toward some passive-film formation during immersion; this is broadly in line with Thiyagarajan et al. [26], who attributed similar surface changes in SS, NiTi, and CuNiTi archwires to oxide-layer formation on contact with saliva. Notably, the surface states recorded after clinical use in the present study did not resemble those seen after short-term saliva immersion, suggesting that immersion testing alone may not capture the changes associated with intraoral aging. For the SS archwire specifically, the comparatively lower impedance magnitude recorded after clinical use, despite closely overlapping OCV trajectories with the as-received state, may point to some thinning or non-uniformity of the passive film. This would be broadly consistent with reports that SS archwires tend to show higher corrosion current densities than NiTi, attributed to a less compact chromium-oxide layer [17], and with earlier work describing a comparatively larger pH-related drop in pitting potential for SS than for NiTi [19]. Suárez et al. [34], testing a range of lingual archwire alloys, similarly reported lower pitting potentials and more extensive polarization damage for SS archwires than for the titanium-based alloys tested, broadly in keeping with the relatively greater surface change observed for SS here. A less stable SS passive film after clinical use could plausibly have some bearing on galvanic interactions with dissimilar bracket alloys [27] and is broadly consistent with reports of increased surface roughness for SS relative to NiTi-based alloys after acidic exposure [35]; however, these comparisons remain indirect and would need to be tested directly.

The NiTi specimen showed pronounced changes relative to the other archwire specimens examined. The deep, slowly recovering OCV transient recorded for the clinically used specimen, together with SEM evidence of features compatible with localized pitting, may be compatible with some disruption of the TiO_2_-rich passive film generally considered central to NiTi’s corrosion resistance [17]. Huang [19] similarly reported that, while NiTi’s TiO_2_-based film gives it a lower passive current density than SS, its pitting potential decreases comparatively little with decreasing pH relative to SS, underscoring the importance of this oxide layer to NiTi’s behavior under more aggressive conditions; incomplete OCV recovery within 900 s further suggests that repassivation after clinical exposure may be slow and that a compromised passive film could leave the clinically used archwire more susceptible to galvanic effects when coupled with dissimilar bracket alloys [27]. Cioffi et al. [36], using a radiotracer method, found that fluoride exposure, rather than mechanical strain, was the main driver of nickel release from NiTi wire via disruption of this same TiO_2_ layer; the present findings may represent a broadly analogous outcome reached through the cumulative chemical and mechanical conditions of clinical service, though this comparison remains speculative given the different experimental conditions. Some of the variabilities observed for NiTi could also reflect differences in phase composition as follows: Briceño et al. [37] reported that a higher martensite content in NiTi archwires was associated with improved corrosion resistance and lower nickel release relative to more fully austenitic wires. Phase composition was not characterized for the archwires in the present study and its possible contribution cannot be excluded.

CuNiTi showed comparatively modest differences between the used and unused conditions, which may suggest some stability associated with copper incorporation. This is tentatively consistent with our own earlier observation of comparatively low nickel release for CuNiTi relative to NiTi [28] and with the relatively limited effect of saliva exposure on CuNiTi reported by Thiyagarajan et al. [26]. A brief OCV transient was nonetheless observed after clinical use, smaller than that of NiTi, suggesting this apparent stability may not be absolute. Further characteristics of CuNiTi archwires have been reviewed elsewhere [38].

The multiforce archwire showed a different pattern from the other alloys tested: EIS suggested a more stable state after clinical use than after saliva immersion in this specimen, the reverse of the trend seen for the other archwires. This may relate to its multi-zone design and the more complex mechanical loading and biochemical exposure occurring intraorally, conditions that a static room-temperature immersion protocol may not reproduce; this interpretation is offered tentatively and would benefit from further investigation. It is broadly in line with the general caution raised by Pakshir et al. [17] which simplified that in vitro electrolyte systems may not fully predict alloy behavior under clinical conditions.

The SEM observations were broadly consistent with the electrochemical trends described above for each archwire type, with greater surface heterogeneity or deposit accumulation tending to coincide with lower measured impedance. For NiTi specifically, the smoother surface of the saliva-immersed specimen relative to the clinically used specimen showing features compatible with pitting is consistent with a protective role for the TiO_2_ layer, a mechanism also supported indirectly by nanocoating work showing that TiO_2_ nanoparticle coatings can reduce nickel–ion release [39].

### Limitations

When interpreting these findings, several methodological considerations should be kept in mind. The present study was designed as a qualitative, comparative assessment of corrosion behavior under defined experimental and clinical conditions rather than as a quantitative or statistically powered analysis. The archwires reflected clinically relevant periods of use, while the in vitro exposure period represented a shorter, controlled model rather than a direct reproduction of clinical aging. Clinically retrieved archwires underwent standardized cleaning and disinfection before analysis, which may have influenced their surface condition. The findings are based on individual measurements and SEM micrographs with no EDS/elemental corroboration, interpreted comparatively and with reference to the literature; further studies incorporating repeated measurements and phase-composition characterization would be valuable for more quantitative confirmation. The study was not blinded.

## 5. Conclusions

The present study demonstrates that the corrosion behavior of nickel-containing orthodontic archwires cannot be adequately characterized by evaluating unused specimens in artificial media alone. In the specimens examined here, in vivo aging introduced surface states that differed from both the as-received and in vitro-immersed conditions, and the direction of that difference appeared archwire-type-specific.

Across all four archwire types, the combined electrochemical (CV, OCV, and EIS) and morphological (SEM) data support the following plausible archwire-specific conclusions:

(a) Stainless steel (SS) archwire exhibited a reduction in impedance response following eight weeks of clinical use, with SEM showing features compatible with the accumulation of granular deposits along pre-existing striations. These findings are consistent with progressive passive-film degradation during intraoral service and suggest that, in the specimen examined here, the clinically aged SS archwire may present a greater risk of galvanic corrosion when coupled with dissimilar bracket alloys than its as-received counterpart; this observation requires confirmation in replicated studies.
(b) The NiTi archwire specimen showed pronounced surface changes relative to the other archwire types studied. The deep and slow-recovering OCV transient of the clinically used archwire, corroborated by SEM evidence of features compatible with discrete pitting, is compatible with eight weeks of intraoral use compromising the integrity of the TiO2-rich passive film that underpins NiTi corrosion resistance.
(c) In the specimens studied here, the CuNiTi archwire showed comparatively stable electrochemical behavior among the NiTi-family archwires examined, with a smaller difference in surface state between the used and unused conditions than observed for the other NiTi-family specimens. In this specimen, copper incorporation may be associated with greater stability of the oxide layer against in vivo degradation; this observation requires confirmation in replicated studies.
(d) The multiforce archwire specimen exhibited an inverse aging pattern relative to the other archwire types examined as follows: clinical use, which encompasses mechanical loading across three distinct force-delivery zones, thermal cycling, and salivary protein exposure, was associated with higher rather than lower corrosion resistance relative to the as-received state in this specimen, a response that static immersion in artificial saliva at room temperature did not replicate. This finding underscores that simplified in vitro protocols may be insufficient for predicting the in vivo behavior of structurally complex, multi-zone archwire architectures.

Taken together, these findings lead to these broad conclusions:

(a) In vitro artificial saliva immersion may not be a reliable surrogate for in vivo aging.
(b) The inclusion of clinically used orthodontic archwires, alongside unused and in vitro-treated specimens, can be helpful for establishing an accurate corrosion profile of orthodontic archwires.

## Figures and Tables

**Table 1:** Clinical use periods for the examined archwires.

Archwire	Period of Use
Stainless steel (Ortho-byte, Wilmington, DE, USA)	8 weeks and 4 days
Superelastic nickel–titanium (Ortho-byte, Wilmington, DE, USA)	8 weeks and 2 days
Copper–nickel–titanium thermodynamic (Ormco, CA, USA)	8 weeks and 6 days
Multiforce NiTi (GC Orthodontics Europe GmbH, Breckerfeld, Germany)	6 weeks and 3 days *

* The differing period of use for the multiforce archwire was chosen based on previous work [28]. In that study we found that this archwire type exhibits a peak in nickel release around week 4, followed by a drop and stabilization, and thus may be effective for treatments of up to 4–6 weeks. Based on that study the treating orthodontist determined 6 weeks to be an appropriate length of treatment with this archwire type.

## Data Availability

The original contributions presented in this study are included in the article. Further inquiries can be directed to the corresponding author.
